# Analytical assessment of the intense heat load of whipping cream, coffee cream, and condensed milk at retail in Austria and Germany

**DOI:** 10.1007/s13594-016-0295-0

**Published:** 2016-06-17

**Authors:** Lisa I. Boitz, Helmut K. Mayer

**Affiliations:** Department of Food Science and Technology, Food Chemistry Laboratory, BOKU, University of Natural Resources and Life Sciences Vienna, Muthgasse 11, A-1190 Vienna, Austria

**Keywords:** Furosine, Lactulose, β-Lactoglobulin, Cream, Condensed milk

## Abstract

Time temperature integrators (TTIs) are useful tools in estimating the heat load applied on differently processed dairy products. The objective of this study was to analyze and assess three TTIs – lactulose, furosine, and acid-soluble β-lactoglobulin (β-Lg) – in 70 high heated dairy products at retail in Austria and Germany comprising whipping cream, coffee cream/milk, and condensed milk products. While β-Lg was not appropriate to evaluate the heat load of these products, furosine and especially lactulose increased with rising intensity of heat treatment, and are appropriate to distinguish between several heating categories analyzed. Pasteurized (*n* = 8) and “heat treated” (*n* = 5) whipping cream samples showed lowest furosine (48 ± 14/ 45 ± 19 mg.100 g^−1^ protein) and low lactulose (29 ± 10/57 ± 28 mg.L^−1^) concentrations, followed by ESL whipping cream (*n* = 10), ESL coffee cream (*n* = 1), and UHT whipping cream (*n* = 10) (furosine = 72 ± 37/71/161 ± 30 mg.100 g^−1^ protein; lactulose = 56 ± 41/161/195 ± 39 mg.L^−1^), respectively. Sterilized condensed milk samples (*n* = 14) showed the highest concentrations of both TTIs and could be clearly separated from UHT treated samples (*n* = 5) (furosine = 491 ± 196/216 ± 46 mg.100 g^−1^ protein; lactulose = 1997 ± 658/409 ± 161 mg.L^−1^), whereas the so-called heat-treated samples (*n* = 9) had a heat load in between showing an extreme range of variation for both TTIs.

## Introduction

Although bovine liquid milk is the major dairy product consumed with 77 kg per capita and year in Austria, these commodities also include whipping cream, coffee milk or coffee cream, evaporated or condensed milk as well as milk powder (AMA 2014). Moreover, Extended Shelf Life (ESL) products have gained widespread acceptance in Austria and Germany; even “ESL” labeled coffee milk came on the market in Austria recently. The Austrian Food Codex defines “cream” as a product with at least 10% milk fat content, *coffee cream* contains 10–18% milk fat, *whipping cream* at least 30%, and a fat content of 36% is necessary for products labeled as “*extra creamy*” whipping cream (FMH 2011). Condensed or concentrated milk products require an additional evaporation step, commonly implemented in falling film evaporators, thereby increasing its milk solids and viscosity. Due to the same production technologies, and to simplify the following text, the term “condensed milk” includes also concentrated dairy products.

**Fig. 1:**
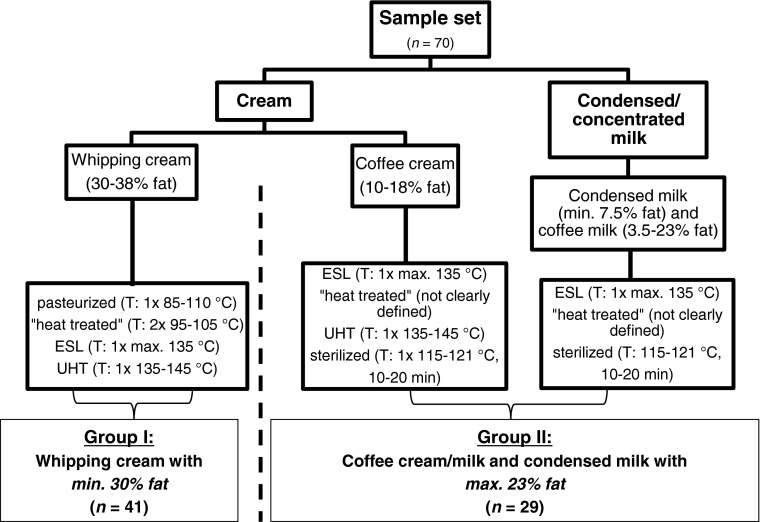
Classification of sample set according to their production technology, fat content, and applied heat treatment as well as chosen arrangement into “group I” and “group II” for data evaluation. “1x”– single flow heating; “2x”– second heating prior to filling

After homogenization and adjustment of fat content, a heat treatment step is necessary to minimize microbial spoilage, inactivate pathogens, and to guarantee a certain shelf life of the product depending on the heat load applied. Table 1 compares processing conditions of pasteurized *milk* to those applied to various types of *whipping cream* and sterilized *coffee cream*/*milk*. While the label “ESL” for whipping cream is not commonly used in Germany, the label “heat treated” (“wärmebehandelt”) could be often found on whipping cream packaging indicating a second heating step using temperatures between 95 and 105 °C of whipping cream prior to filling (indicated with “2x” in Fig.1).

**Table 1:** Processing conditions of pasteurized milk and various types of whipping cream and sterilized coffee cream/milk as well as their shelf life (FMH 2011; LVBM 2015)

Product	Processing conditions	Shelf life and storage
Pasteurized milk	≤75 °C, few seconds	10 days, chilled
Pasteurized whipping cream	Austria: 85–110 °C Germany: 95–105 °C	10 days, chilled
ESL whipping cream (Austria)	≤135 °C, single flow	21 days, chilled
UHT whipping cream	135–150 °C, few seconds	6 months, ambient conditions
Sterilized coffee cream/milk	In-container sterilized: 121 °C, 20 min	1 year, ambient conditions

It is already known and well studied that different time/temperature combinations applied during processing according to the product segment (pasteurization/ESL/UHT or sterilization) can lead to enormous chemical (e.g., Maillard reaction products), nutritional (e.g., vitamin loss), and/or sensorial (e.g., cooked flavor) differences in the final dairy products. To assess the impact of a thermal process on dairy products, intrinsic time temperature integrators (TTIs) provide a useful tool for the quantification of the heat load of liquid milk (Claeys et al. 2002). They are categorized as follows: type I indicators include denaturation, degradation, and inactivation processes of heat labile components, most suitable for the evaluation of low-heat treatments, e.g., enzymes, water- and fat-soluble vitamins, and β-lactoglobulin (β-Lg). Type II indicators describe the formation of substances that are (almost) not present in raw milk, including lactulose, 5-hydroxymethylfurfural, and furosine, and are more appropriate for the assessment of high-temperature processes (Mayer et al. 2010).

Unfolding of the heat sensitive protein β-Lg, comprising 55% of total whey protein in bovine milk, occurs above 60 °C. Thus, the remaining concentration of native acid-soluble β-Lg observed after thermal processing reveals a suitable TTI to assess the heat load of liquid milk (Manzo et al. 2015). For rapid screening, electrophoretic methods are commonly used (Mayer et al. 2010), and for an exact quantification, the IDF recommends a reference HPLC method (ISO/IDF standard 178, 2005).

Furosine (ε-*N*-[2-furoylmethyl]-l-2-lysine), produced by acid hydrolysis of lactulosyllysine, is related to the first stages of Maillard reaction which is associated with the production of every heated dairy product, while its extent depends on the applied time/temperature conditions during processing, especially above 120 °C (Töpel 2007). Due to a positive correlation of furosine concentration and total protein content of the product, the final furosine concentration is given in relation to 100 g protein. Evaluation of furosine in milk is mostly accomplished with HPLC methods, as supposed by the ISO/IDF standard 193 (2004) or other research groups (Mayer et al. 2010). Additionally, lactulose as a further type II indicator was to be determined. This disaccharide comprising fructose and galactose is formed by lactose isomerization, and is a result of the so-called Lobry de Bruyn–Alberda van Ekenstein transformation. Lactulose is not detectable in raw milk, and lowest amounts are found in pasteurized milk, with increasing concentrations with increasing heat load applied (Mayer et al. 2010; Montilla et al. 2005). For quantification, a HPLC method (ISO/IDF standard 147, 2007) and further methods were reported in the literature, e.g., enzymatic determination (Amine et al. 2000).

Intensive research was accomplished considering several TTIs in liquid milk. Furosine and lactulose concentrations increase with rising heat load applied to liquid milk (Gallmann et al. 2001; Mayer et al. 2010; Montilla et al. 2005). However, information on further dairy products besides liquid bovine milk is rare. Some literature considering lactulose contents in high-temperature treated dairy products, such as condensed milk, is available. Nevertheless, these studies are outdated or do not reflect currently used dairy technologies (Adachi and Patton 1961), included only a limited sample set for condensed/sterilized products (Montilla et al. 2005), or did either not consider other TTIs to differentiate the various heating categories of dairy products or were only analyzed in liquid milk or milk powder and not in higher heated dairy products (Sakkas et al. 2014). Hence, the aim of this study was to evaluate appropriate TTIs for dairy products which are generally processed at higher temperatures than liquid milk.

As we have already shown recently (Boitz and Mayer 2015; Mayer et al. 2010), β-Lg as type I indicator is a suitable heat load indicator for liquid milk, but definitely not suitable for the heat load assessment in dairy products with a more intense heat treatment, like whipping cream. Thus, the assumption that β-Lg is also not appropriate as heat load indicator for even higher heated products than whipping cream, such as condensed milk, was to be confirmed in this study. Furthermore, the aim of this study was to determine two different type II indicators, namely lactulose and furosine, in several dairy product segments comprising an overall higher heat load during its production compared to pasteurized liquid milk. Thus, the purpose of our work was to provide novel information on three TTIs for high heated dairy products, which is not available in the present literature. Within the product segments “whipping cream” and “coffee cream/milk and condensed milk”, the different heating categories “heat treated”, “ESL”, “UHT”, and “sterilized” were intensively studied. Finally, it was to be proven if lactulose and furosine are suitable TTIs for these dairy product segments as well.

## Materials and methods

### Chemical references

All solvents used for chromatographic analysis were of HPLC grade, and all chemicals used for sample preparation were of analytical grade. Throughout all experiments, aqueous solutions were prepared with ultrapure water (SG Ultra Clear UV system; Siemens Water 95 Technologies, Warrendale, PA, USA). Acetonitrile (ACN, 100%), disodium hydrogen phosphate (99%), heptanesulfonic acid (>98%), hydrogen peroxide (30%), magnesium sulfate heptahydrate (98%), sodium acetate trihydrate (99.5%), sodium dihydrogen phosphate, octanol (98%), and trifluoroacetic acid (TFA, 99%) were purchased from Sigma-Aldrich (St. Louis, MO, USA). Furosine standard (furosine dihydrochloride; >97%) for calibration was purchased from NeoMPS PolyPeptide Laboratories Group (Strasbourg, France). For native PAGE, ammoniumpersulfate (>99%) and tetramethylethylendiamine were purchased from Serva (Heidelberg, Germany), and bromophenol blue as well as glycine were purchased from Merck (Darmstadt, Germany).

For the determination of lactulose, zinc sulfate (99.5%) and potassium hexacyanoferrate (II) (99.0%), required for Carrez solution I and II, were purchased from Merck (Darmstadt, Germany), and triethanolamine (TEA, 99.0%) buffer was obtained from Acros Organics (Geel, Belgium). Sodium hydroxide (NaOH, 99%), hydrochloric acid (HCl, 37%), and formic acid (FA, 98%) were purchased from Roth (Karslruhe, Germany). Enzymatic determination of lactulose was accomplished with ready-to-use solutions and enzymes included in the commercial enzyme kit (K-Lactul; Megazyme, Bray, Co. Wicklow, Ireland). Solutions and enzymes were stored as prescribed in the manual, and frozen solutions were aliquoted to avoid unnecessary thawing processes.

### Dairy product sample set

The sample set included 70 dairy product samples of different processing categories: whipping cream, coffee cream/milk, and condensed milk. Figure 1 shows a classification of sample set according to the production and the related technology as well as to the respective differentiation into “group I” and “group II.” Nutritional values were either taken from the label (carbohydrates) or determined in the laboratory (fat and protein content). All commercial whipping cream samples (group I, *n* = 41) produced by various dairy companies using different heating technologies were obtained from retail outlets either in Austria or in Germany. These samples included previously analyzed samples: 9 pasteurized, 13 ESL (2 lactose-free), and 11 UHT (2 lactose-free) whipping creams as described in Boitz and Mayer (2015), and additionally, 2 UHT samples and 6 German whipping cream samples were labeled as “heat treated.” These samples contained 29.50–38.33% fat, 1.76–2.59 g.100 g^−1^ protein, and 3.0–3.4 g.100 g^−1^ carbohydrate. Condensed milk and coffee cream/milk samples (group II, *n* = 29) included 14 samples labeled as “coffee cream” (*n* = 13) or coffee milk (*n* = 1) and 2 samples labeled directly as “concentrated coffee milk”. The fat content was between 3.50 and 15.00 g.100 g^−1^, the protein content was between 2.73 and 6.43 g.100 g^−1^, and the carbohydrate content was between 3.8 and 10.0 g.100 g^−1^. Group II also included 13 samples labeled as “condensed milk” (eight unsweetened, five sweetened) with a fat content of 7.50–23.00 g.100 g^−1^, a protein content of 2.57–7.52 g.100 g^−1^, and a carbohydrate content of 7.6–55.0 g.100 g^−1^. All condensed/concentrated products showed an increased fat-free milk dry matter ranging from 12.6 to 20.0%. According to their production process and related heat treatment, samples of group II can be further subdivided into the only available ESL sample, 9 samples labeled as “heat treated” (produced either in Germany or in Austria), 5 UHT, and 14 sterilized samples (including the five sweetened condensed milk samples). All samples were analyzed within their labeled shelf-life period (and stored at 4 °C until first analysis). Long-life products were analyzed within the first quarter of the time period given as “date of expiry”. In case of coffee cream samples which were packed in small-sized plastic cups (10 g, respectively), all cups from the sample package were pooled, yielding approximately 200 g of sample. Prior to analysis, all samples were warmed in a water bath set at 40 °C, and this temperature was held for 15 min to get a homogenous emulsion. Samples were aliquoted and kept frozen until further analyses. Unless otherwise specified, all analyses were carried out in duplicate.

### Type II indicator furosine: RP-HPLC analysis

Determination of furosine in all dairy samples was accomplished according to ISO/IDF (2004) with some modifications as described previously (Boitz and Mayer 2015). Due to the high viscosity of some condensed milk samples, a pre-dilution was necessary (*w*/*w*). Afterwards, 2 g of each sample was mixed with 6 mL 10.6 mol. L^−1^ HCl in screw-cap Pyrex® tubes and exposed to nitrogen for 2 min. For acid hydrolysis, the tubes were closed tightly and heated at 110 °C for 23 h. After heating, the tubes were cooled at ambient conditions, and hydrolysates obtained were filtered (Schleicher & Schuell 595 ½; GE Healthcare, Buckinghamshire, UK) and kept frozen until further analysis. After solid phase extraction of 0.5 mL sample hydrolysate using Waters SepPak® Vac 3cc (500 mg) Certified Silica Cartridges (Waters Corporation, MA, USA), eluates were vacuum dried in a Pico·Tag workstation (Millipore Waters Workstation PTS 3142, pump BOC EDWARDS, model XDS5). Dried samples were diluted in 0.2 mL of freshly prepared diluting solution consisting of 5% ACN and 0.2% FA in ultrapure water. Samples were filtered (0.2 μm, Minisart RC4; Sartorious, Goettingen, Germany) prior to HPLC injection. Furosine detection was performed on a Waters chromatography system using a model 600E multisolvent delivery system, a Rheodyne 7725i injector, guard column (Sentry Guard, Symmetry™ C_18_, 3.5 μm, 2.1 × 10 mm), and a Symmetry™ 300 C_18_ column (3.5 μm, 2.1 × 150 mm) (Waters Corporation, MA, USA). The isocratic separation (flow rate 0.35 mL.min^−1^; injection volume 20 μL; column temperature 35 °C) was accomplished with 89% of solvent A consisting of 5 mM heptanesulfonic acid with 0.2% FA and 11% of solvent B (100% ACN). Eluates were detected at 280 nm using a Waters 2489 UV/VIS Detector which was interfaced with a PC running Waters Millennium^32^ chromatography manager. The calibration was performed by plotting peak area versus picomoles of furosine and was linear (*R*^2^ = 0.999) between 5 and 60 pmol per injection. Inter-day repeatability of sample preparation procedure (same analyst and apparatus but different reagents) was verified with eight independent sample work-ups of pasteurized whipping cream (35 mg.100 g^−1^ protein) and UHT whipping cream (155.2 mg.100 g^−1^ protein) samples with resulting RSDs of 2.9 and 1.5%, respectively.

Total nitrogen content required for the final furosine calculation of samples was analyzed in triplicate according to the Kjeldahl method (ISO/IDF 2001). Further, the total fat content of samples was determined according to Gerber’s or Roeder’s butyrometric method (DIN standard 10284: 1964-03; ISO/IDF 2008).

### Type II indicator lactulose: enzymatic determination

Lactulose concentrations were determined enzymatically because of economical reasons and its easy handling with a modified test kit method (K-Lactul; Megazyme, Bray, Co. Wicklow, Ireland) as described recently (Boitz and Mayer 2015). Chromatographic methods were not necessary due to satisfying precision and sensitivity of used enzyme kit for samples with an expected high concentration of lactulose in the analyzed samples. Briefly, samples were defatted by double centrifugation (first = 20 min, 17,200×*g*, 4 °C; second = 3 min, 15,700×*g*, 4 °C), and after dilution of obtained fat-free phase, the samples were mixed carefully with sodium phosphate buffer (0.4 mol.L^−1^, pH 7.6) and ultrapure water. After clarification with Carrez solutions I and II, the supernatant was transferred into tightly closing screw-cap microfuge tubes (SC Micro Tube PCR-PT, Sarstedt, Germany) due to intense pressure development during enzymatic reaction in the reaction tube. Each sample required a separate blank, containing water instead of the enzyme β-galactosidase, which was added to the sample hydrolyzing both lactose and lactulose to galactose as well as glucose and fructose, respectively. After the addition of sodium acetate buffer (0.5 mol.L^−1^, pH 4.5), tubes were incubated 60 min at 40 °C in a covered thermoblock. Afterwards, TEA buffer (1 mol.L^−1^, pH 7.6), NaOH (0.33 mol.L^−1^), octanol, and enzyme solution (catalase/glucose-oxidase) as well as hydrogen peroxide were added, followed by another incubation step for 15 min at 40 °C and a subsequent centrifugation (10 min, 15,700×*g*, 20 °C) terminating the enzymatic reactions. After further enzymatic treatment as described in the enzyme kit manual, the absorbance of released NADPH was measured at 340 nm using a UV/VIS spectrophotometer (U-2000; HITACHI, Chiyoda, Japan). The final lactulose concentration (mg.L^−1^) was calculated from measured lactulose in the fat-free phase considering the fat concentration analyzed for each sample. Referring to lactose-free or artificially sweetened products, the high concentration of free fructose in the sample led to further enzymatic reactions in the blank as well, making the kit unsuitable for these products. Thus, artificially sweetened and lactose-free samples included in the sample set were not included in further evaluation of results considering lactulose.

### Type I indicator β-Lg: estimation by native PAGE of samples

Due to the assumption of a β-Lg concentration in high-temperature treated dairy products (especially coffee cream/milk and condensed milk samples) lower than the LOQ (20 mg.L^−1^) of the used RP-HPLC method, a fast and easy electrophoretic technique was chosen to prove this assumption. Native polyacrylamide gel electrophoresis (native PAGE) was used to analyze acid-soluble β-Lg in selected samples separating the whey proteins by their different electrophoretic mobility (according to their electrical charge) during migration towards the positive pole (anode). Precipitation of caseins (including also the heat-denatured whey proteins) was accomplished by acidification of samples. Therefore, the homogenous samples were defatted by centrifugation (15 min, 4 °C, 6450×*g*), and HCl (2 mol.L^−1^) was added carefully, until a pH of 4.5 was reached. Samples were let stand at ambient conditions and centrifuged again (30 min, 4 °C, 17,200×*g*). The acid whey (supernatant) was aliquoted and kept frozen until further analysis. Sample preparation included a tempering step (10 min, 40 °C) of 100 μL acid whey and the subsequent addition of 400 μL buffer (10 mmol.L^−1^ tris (hydroxymethyl)-aminomethane and 77 mmol.L^−1^ glycine, pH 8.3) as well as 55 μL of bromophenol blue solution. The sample was centrifuged for 5 min (16,100×*g*), and 20 μL of the supernatant was used for electrophoretic separation. Native PAGE (12.5% T) was performed using a dual cooled vertical slab gel electrophoresis unit SE 600 (Hoefer Scientific Instruments, San Francisco, CA, USA) as described in Mayer et al. (2003).

### Type I indicator β-Lg: quantification by RP-HPLC analysis of β-lactoglobulin in whipping cream samples

In order to determine the accurate β-Lg concentration in all whipping cream samples and in some selected group II samples (having visible β-Lg bands in electrophoretograms), these samples were analyzed with RP-HPLC as described previously (Boitz and Mayer 2015).

Briefly, after acid precipitation of caseins (and denatured whey proteins) with HCl (2 mol.L^−1^, pH 4.5), the obtained acid whey was diluted (1:10) with phosphate buffer (0.1 mol.L^−1^, pH 6.7), incubated for 1 h, and samples were filtered prior to HPLC injection (0.2 μm, Minisart RC4; Sartorious, Goettingen, Germany). The same RP-HPLC column and equipment was used as described for the determination of furosine above as well as in Boitz and Mayer (2015). A gradient separation was performed with solvent A consisting of 0.1% TFA in ultrapure water, and solvent B consisting of 0.1% TFA in ACN, using a column temperature of 40 °C and a flow rate of 0.35 mL.min^−1^. UV/VIS detector was set at 205 nm, and injection volume was 10 μL. The calibration was linear (*R*^2^ = 0.998) between 0.2 and 3.2 μg per 10 μL injection volume which is equivalent to a β-Lg content of 0.2–3.2 mg.mL^−1^ in liquid milk after the performed dilution (1:10). Considering inter-day repeatability of sample preparation procedure (same analyst and apparatus but different reagents), a RSD of 3.4% was determined for an independent sample work-up (*n* = 8) of a UHT whipping cream sample (130 mg.L^−1^).

### Statistical analysis of results

Calculations of mean values and standard deviations as well as related artworks were accomplished with Microsoft Excel 2010. Moreover, data on lactulose and furosine concentrations were analyzed by one-way ANOVA and post-tested by Tukey test using SPSS 21.0 software, and differences were considered significant at *p* <0.05.

## Results

### RP-HPLC analysis of furosine

**Fig. 2:**
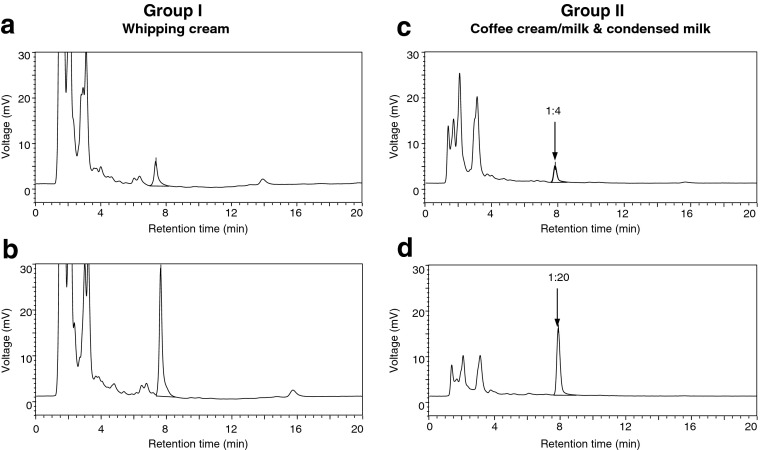
RP-HPLC chromatograms (*λ* = 280 nm) of furosine in whipping cream samples (group I) on the left side (**a** pasteurized–52.9 mg. 100 g ^−1^ protein, **b** UHT–176.6 mg. 100 g ^−1^ protein) and in condensed milk/coffee cream samples (group II) on the right side (**c** ESL–70.9 mg. 100 g ^−1^ protein, **d** sterilized–789.1 mg. 100 g ^−1^ protein), respectively

Seventy retail samples including 41 whipping cream samples (for furosine data of pasteurized and ESL samples; see Boitz and Mayer 2015) and 29 coffee cream/milk and condensed milk samples were analyzed for their furosine concentrations using an existing RP-HPLC method with few modifications in sample preparation because of higher fat content and higher viscosity, especially of condensed milk samples. Figure 2 shows chromatograms of two examples of differently processed whipping cream samples (group I) as well as two examples of coffee cream/milk and condensed milk samples (group II), respectively. While no (or only a slight) dilution was necessary for proper detection of whipping cream samples (Fig. 2a, b), coffee cream/milk samples and condensed milk samples had to be diluted to avoid an overload of the HPLC column (Fig. 2c, d). Nevertheless, for the undiluted whipping cream samples, peak area increased with increasing intensity of heat treatment. The “ESL” coffee cream sample showed the smallest furosine peak and lowest amount within group II (Fig.2c; 70.9 mg.100 g^−1^ protein) and could be clearly separated from all other samples in group II.

**Fig. 3:**
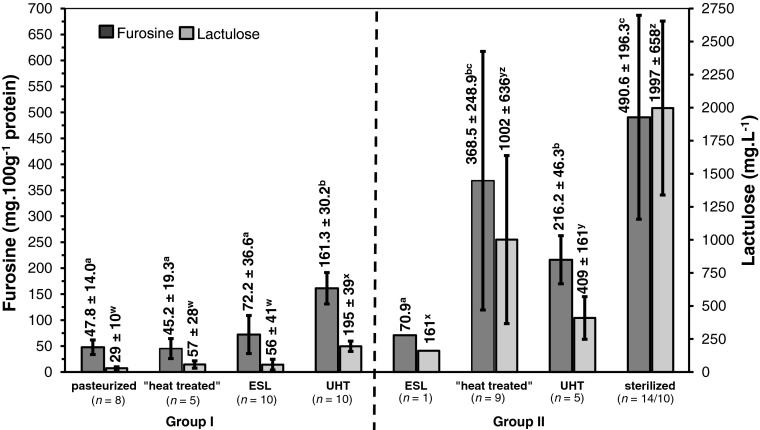
Heat load indicators furosine and lactulose (mean value ± SD) of whipping cream samples (group I, left side), and coffee milk/cream and condensed milk (group II, right side) according to their heat load category. Lactose-free samples and abnormally high values were not included. Different *superscripts* indicate differing least square means (*p* < 0.05) for furosine (^abc^) or lactulose (^wxyz^), respectively. Data for pasteurized and ESL whipping cream samples of group I have been already reported recently (Boitz and Mayer 2015)

Figure 3 shows mean values and standard deviations of the analyzed furosine and lactulose concentrations for pasteurized, ESL, “heat treated”, and UHT whipping cream (group I, left side) as well as for ESL, “heat treated”, UHT, and sterilized coffee cream/milk and condensed milk samples (group II, right side), respectively. Lowest furosine concentrations were found in pasteurized whipping cream samples (47.8 ± 14.0 mg.100 g^−1^ protein), and increasing concentrations were detected with rising processing temperatures in ESL samples (72.2 ± 36.6 mg.100 g^−1^ protein) and UHT treated samples (161.3 ± 30.2 mg.100 g^−1^ protein), respectively. German whipping cream samples labeled as “heat treated” showed quite similar furosine concentrations (45.2 ± 19.3 mg.100 g^−1^ protein) as pasteurized whipping cream samples, but lower values than ESL whipping cream samples. Referring to group II and when comparing mean values of “heat treated” coffee cream/milk and condensed milk samples (368.5 ± 248.9 mg.100 g^−1^ protein) to UHT samples (216.2 ± 46.3 mg.100 g^−1^ protein), “heat treated” samples showed higher furosine concentrations than UHT coffee cream/milk samples. However, “heat treated” coffee cream/milk and condensed milk samples showed more than twice the amount of UHT whipping cream samples of group I (161.3 ± 30.2 mg.100 g^−1^ protein). Interestingly, “heat treated” samples of group II revealed a wide standard deviation of furosine concentrations (368.5 ± 248.9 mg.100 g^−1^ protein). Lowest furosine amount was found in the single ESL sample analyzed with 70.9 mg.100 g^−1^ protein, and highest amounts were found in sterilized coffee cream/milk and condensed milk samples (490.6 ± 196.3 mg.100 g^−1^ protein). As seen in Fig. 3 and confirmed by statistical analysis, sterilized coffee cream/milk samples differed significantly from all other groups (*p* < 0.001), with exception of “heat treated” coffee cream/milk samples. These samples could be clearly distinguished from pasteurized, “heat treated,” and ESL whipping cream samples (*p* < 0.002), respectively.

### Enzymatic determination of lactulose

All samples were analyzed for their lactulose concentrations in their obtained fat-free phase, and the final lactulose concentration was calculated according to their corresponding fat content (for lactulose data of pasteurized and ESL samples; see Boitz and Mayer 2015). Since the enzymatic determination of lactulose includes a cleavage of disaccharides, the lactulose concentration could not be measured precisely in industrial sweetened products (*n* = 5), and consequently, these samples were not included into the calculation of mean values for lactulose in sterilized samples. Figure 3 shows increasing amounts of lactulose with rising heat load of dairy products. The lowest amounts were found in pasteurized whipping cream samples (29 ± 10 mg.L^−1^), followed by “heat treated” (57 ± 28 mg.L^−1^) and ESL labeled whipping cream samples (56 ± 41 mg.L^−1^). The highest amounts within group I were found in UHT whipping cream samples (195 ± 39 mg.L^−1^). A slightly lower amount was found in the single ESL coffee milk sample analyzed (group II, 161 mg.L^−1^). UHT samples of group II showed more than twice the amount (409 ± 161 mg.L^−1^) of the ESL coffee milk (161 mg.L^−1^), and interestingly, the “heat treated” samples of group II had approximately a six times higher amount (1002 ± 636 mg.L^−1^) compared to the ESL sample. As expected, in sterilized samples, even a 12 times higher amount (1997 ± 658 mg.L^−1^) was found. As for furosine, also lactulose results showed a wide variation especially referring to “heat treated” and sterilized samples of group II (Fig. 3).

### β-Lactoglobulin analysis of samples

**Fig. 4:**
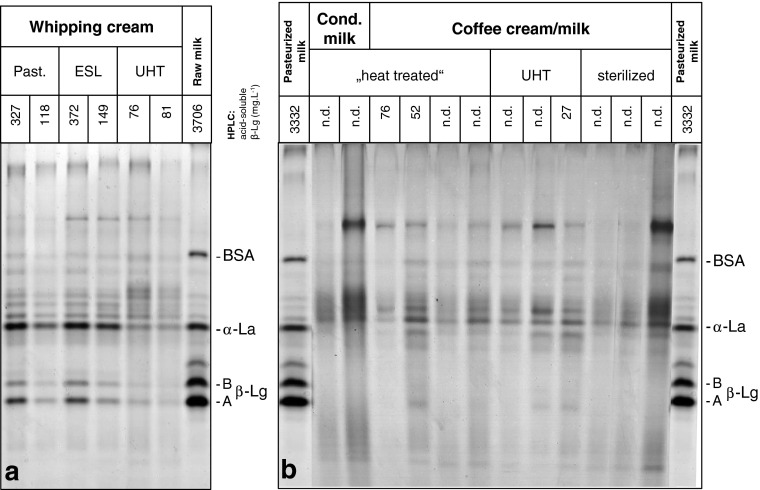
Native polyacrylamide gel electrophoresis (12.5% T) of whey protein fractions soluble at pH 4.6 of different dairy products. **a** Whipping cream samples (group I, left side): pasteurized, ESL, and UHT samples; **b** selected coffee cream/milk and condensed (cond.) milk samples (group II, right side): “heat treated”, UHT, and sterilized samples. Raw milk and pasteurized milk are given as references. BSA (bovine serum albumin), α-lactalbumin (α-La), and β-lactoglobulin (β-Lg) as well as β-Lg concentrations as determined with RP-HPLC in milligrams per liter (if concentration was under LOQ–*n.d.* not determined)

Selected whipping cream, coffee cream/milk, and condensed milk samples of different heating categories (pasteurized, “heat treated”, ESL, UHT, sterilized) were applied to native PAGE (12.5% T). Acid-soluble whey proteins were separated, and the results of both groups are shown in Fig. 4a and b, where raw milk and pasteurized milk are given as references. Protein patterns of raw milk (Fig. 4a) and pasteurized liquid milk (Fig. 4b) showed intense bands of typical whey protein fractions (e.g., α-La, bovine serum albumin (BSA) and β-Lg), whereas banding patterns of whipping cream samples were weaker (especially β-Lg), thereby showing distinct differences in intensity comparing the different heating categories (Fig. 4a). Obviously, intensity of acid-soluble whey proteins decreased corresponding to an increasing heat load of whipping cream samples analyzed. Thus, pasteurized and ESL whipping cream could be clearly differentiated from UHT whipping cream samples; corresponding β-Lg concentrations measured by RP-HPLC are given on top of the electrophoretogram (Fig. 4a).

**Fig. 5:**
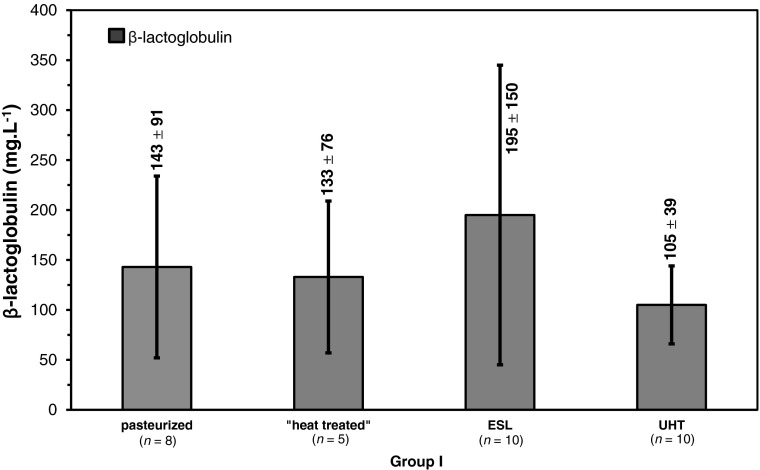
β-Lg concentrations (mean values ± SD in mg.L^−1^) of differently processed whipping cream samples purchased from Austrian and German market. Data for pasteurized and ESL samples have been already reported recently (Boitz and Mayer 2015)

Referring to the acid whey proteins analyzed in group II samples, no β-Lg bands were visible due to excessive heat load, and consequently, no differences in the intensity of β-Lg banding patterns could be observed in these samples (Fig. 4b). Smearing lanes may be a result of concentrated products, although samples were diluted prior to analysis. However, as whipping cream samples could be differentiated by their distinct banding patterns (Fig. 4a), these and some selected samples of group II were analyzed for their accurate β-Lg contents with RP-HPLC. Results for whipping cream samples are shown in Fig. 5, whereas most of the selected samples of group II were below the limit of quantification (LOQ was 20 mg.L^−1^).

## Discussion

In this study, different dairy products suffering a more intense heat treatment in comparison to liquid milk were analyzed according to several heat load indicators. Results obtained by a rapid screening of acid-soluble β-Lg concentrations of selected samples using native PAGE showed obvious differences in band intensity in group I samples (Fig. 4a), but no remaining bands of acid-soluble β-Lg were obtained for group II samples due to excessive heating (Fig. 4b). Thus, this electrophoretic method is not suitable for coffee cream/milk or condensed milk samples due to low β-Lg concentrations. RP-HPLC analysis revealed concentrations of acid-soluble β-Lg below the LOQ of 20 mg.L^−1^ for most of the selected samples of group II, whereas acid-soluble β-Lg was successfully determined in all whipping cream samples of group I (see Fig. 5). The obtained results for group I samples did not show any correlation between the intensity of applied heat load and the corresponding concentration of acid-soluble β-Lg. Concentrations of German whipping cream products labeled as “heat treated” (“wärmebehandelt”), which are heated twice, were comparable to obtained results of the pasteurized whipping cream samples (heated only once), indicating a mild heat treatment for both heating categories. Nevertheless, ESL whipping cream samples showed highest β-Lg amounts with a great variation of results, and the UHT samples (with the most intense heat treatment) showed lowest β-Lg amounts. As shown in Table 1, processing conditions of ESL whipping cream are situated somewhere between 105 and 135 °C, which is close to those practically applied for UHT whipping cream (135–140 °C). Both the wide range of temperatures applied for ESL processing and the fluent transition of ESL and UHT technology may explain the broad variation of results with the highest values for ESL samples (Fig. 5). However, these results highlight the statement as described previously in Boitz and Mayer (2015) that β-Lg is definitely not appropriate as a heat load indicator for whipping cream. As the concentrations were below the LOQ of RP-HPLC method for the majority of group II samples, β-Lg as type I indicator is not suitable for assessing the heat load of high-temperature treated dairy products such as coffee cream/milk and condensed milk, neither for UHT or “heat treated” nor for sterilized samples.

In contrast to this, type II indicators lactulose and furosine indicated a relationship between overall heat load applied and concentration analyzed. The more intense the head load (including heating time and processing temperature), the higher the lactulose and furosine concentrations. As confirmed by statistical tests, coffee cream/milk and condensed milk samples showed much higher lactulose and furosine values in comparison to whipping cream samples in general. Comparing the lactulose mean values of group I to group II, a significant difference exists (*p* < 0.001) between all heating categories of group I compared to the heating categories “heat treated,” UHT, and “sterilized” of group II. This is in contrast to furosine, where UHT whipping cream samples could not be clearly differentiated from “heat treated” samples of group II (compare Fig. 3). Within group II, lactulose and furosine mean values were significantly different between sterilized samples and UHT samples (*p* < 0.001). Thus, the heat load of sterilized coffee cream/milk and condensed milk products is much higher than those of UHT processed samples, whereas the so-called heat-treated coffee cream/milk samples were in between, showing an extreme range of variation for both TTIs. Referring to lactulose and furosine of sterilized samples, variations might be a result of carbohydrate content ranging from 7.6 to 55.0 g.100 g^−1^ due to different evaporation intensities of condensed products in different dairies.

In combination with the acid-soluble β-Lg concentration, lactulose proved to be a useful tool to distinguish between UHT and in-container sterilized milk (European Commission 1992; ISO/IDF 1992, 1993). Thus, the EU and the IDF suggested a maximal lactulose concentration of 600 mg.L^−1^, associated with a minimum β-Lg concentration of 50 mg.L^−1^ for UHT milk (European Commission 1992; ISO/IDF 1992, 1993). In-container sterilized milk shows more than 600 mg.L^−1^ lactulose and less than 50 mg.L^−1^ of acid-soluble β-Lg (Corzo et al. 1996).

In this context, comparing the measured lactulose concentrations, “heat treated” as well as sterilized coffee cream/milk samples showed an amount above 600 mg.L^−1^ and can be clearly distinguished from UHT treated samples showing lactulose concentrations below 600 mg.L^−1^. However, this statement does not apply to the β-Lg concentrations, as UHT coffee cream/milk samples showed values below 50 mg.L^−1^ due to the higher heat load compared to UHT milk. Consequently, the lactulose concentration may indicate a possible differentiation between UHT treated coffee cream/milk and “heat treated” or sterilized products, but β-Lg is not appropriate to distinguish between these categories.

Interestingly, when comparing the UHT treated samples of both groups, the furosine concentration is not significantly (*p* > 0.05) higher in group II samples (216.2 ± 46.3 g.100 g^−1^ protein) compared to group I samples (161.3 ± 30.2 g.100 g^−1^ protein), but more than twice of the lactulose concentration was determined in group II UHT treated samples (409 ± 161 mg.L^−1^) compared to group I samples (195 ± 39 mg.L^−1^). In this case, lactulose appears to be more meaningful than furosine for a successful differentiation of these heating categories. However, although the production technology of whipping cream and coffee cream is quite similar, both TTIs indicate a completely different heat load applied onto these dairy products. The higher concentrations of lactulose and furosine found in group II samples may indicate a much more intense heat load applied to skimmed milk prior to fat adjustment of these products during manufacturing.

Referring to group I, pasteurized and “heat treated” whipping cream samples showed no difference when comparing the furosine concentrations. Once again, lactulose appears to be more meaningful with higher concentrations in “heat treated samples” (57 ± 28 mg.L^−1^) than in pasteurized whipping cream samples (29 ± 10 mg.L^−1^). Higher lactulose concentrations may result from the second heating step during processing of “heat treated” whipping cream. Thus, “heat treated” whipping cream can be placed between pasteurized and ESL samples due to furosine concentrations similar to pasteurized samples and lactulose concentrations comparable to ESL whipping cream. In contrast to these findings, “heat treated” samples of group II showed higher lactulose and furosine mean values than UHT samples. In particular, lactulose concentrations are twice as high in “heat treated” samples compared to UHT samples (1002 ± 636 mg.L^−1^ and 409 ± 161 mg.L^−1^, respectively). This may show that the heat treatment of coffee cream and condensed cream/milk labeled as “heat treated” (and where no regularly definition exists) can be understood and described as a heat treatment between UHT processing and sterilization. Considering furosine concentrations of “heat treated” samples, a carbohydrate content of 7.6–55.0 g.100 g^−1^ may be one reason for broad variation of results. However, the wide variations of results underline the absence of legal requirements considering time/temperature combinations for coffee cream/milk and condensed milk carrying the label “heat treated.” Accordingly, this absence of thresholds or clear regulations (regarding “heat treated”) offers a wide margin/tolerance to all dairy companies to choose completely different time-temperature combinations for these products, leading to different qualities of final products having all the same label “heat treated.”

As reported recently (Mayer et al. 2010), β-Lg content of raw milk is about 4000 mg.L^−1^. A minimum β-Lg content of 2600 mg.L^−1^ for pasteurized milk, of 2000 mg.L^−1^ for high pasteurized milk, and of 50 mg.L^−1^ for UHT milk had been proposed by the European Commission, respectively (European Commission 1992). In this context, UHT whipping cream samples fulfill these requirements, but considering group II, only one sample (76 mg.L^−1^) showed a concentration above this threshold. These results fit to those reported in Feinberg et al. (2006) who found only 44 mg.L^−1^ in indirectly heated UHT milk samples and no β-Lg in sterilized milk, and to those reported in Morales et al. (2000) who found 63.4 mg.L^−1^ in indirectly heated UHT milk and below 10 mg.L^−1^ in sterilized milk samples, respectively.

The obtained results concerning furosine concentrations in condensed milk products are in accordance with Henle et al. (1995) who found concentrations between 340 and 880 mg.100 g^−1^ protein in eight analyzed condensed milk samples, whereas higher furosine concentration (730–836 mg.100 g^−1^ protein) were detected in two samples analyzed by Vallejo-Cordoba et al. (2004). The lower levels obtained in the present study may indicate a more gentle heat treatment of dairy products analyzed as well as advanced production technologies with less heat load applied onto the products. Elliott et al. (2005) reported a furosine concentration of 124 mg. 100 g ^−1^ protein in indirectly heated UHT milk, Sakkas et al. (2014) reported 53.3 mg. 100 g ^−1^ protein in UHT milk. Obtained results in group I and definitely higher furosine levels of group II reflect an overall higher heat treatment for whipping cream and coffee cream/milk compared to liquid milk.

Considering lactulose, results obtained in this study are different compared to those reported in Montilla et al. (2005), who found lactulose concentrations of 207–313 mg.L^−1^ in condensed milk (*n* = 3) and 623–705 mg.L^−1^ in sterilized milk (*n* = 2). Differences may result from their small sample sets and the gas chromatographic method used for determination. Moreover, the authors did not specify the heat treatment of the analyzed condensed milk samples. Morales et al. (2000) reported a lactulose concentration of 1121 mg.L^−1^ for sterilized milk, applying two heating steps with an overall processing time of approximately 17 min. The obtained lactulose levels in this study were higher, showing a broad standard deviation. Varying carbohydrate content of samples and different industrial conditions and dairy plants may explain these differences. UHT heated milk samples showed lactulose concentrations between 120 and 456 mg.L^−1^. Obtained results for group I were within in this range, whereas group II samples showed much higher values due to higher heat load applied (time/temperature parameters).

## Conclusion

In conclusion, it could be successfully shown that β-Lg as type I indicator is definitely not appropriate for the assessment of heat load of whipping cream and coffee cream/milk as well as condensed milk samples. In contrast, type II indicators lactulose and furosine were useful tools to distinguish between several heating categories. Lactulose appeared to be more meaningful and showed greater significant differences between several heating categories in comparison to furosine. Finally, UHT treated whipping cream samples could be clearly discriminated from pasteurized, ESL, and “heat treated” whipping cream with both TTIs. Sterilized and “heat treated” coffee cream/milk and condensed milk products could be clearly differentiated from UHT, as well as from ESL products considering lactulose concentrations. Moreover, it could be shown that the label “heat treated” on whipping cream can be interpreted as a heat treatment between pasteurization and ESL heat treatment. However, referring to coffee cream/milk and condensed milk, the heat load of “heat treated” products was higher than an ultra-high-temperature treatment (with high lactulose and furosine concentrations), and just below the excessive heat load of sterilized products.
